# Identity-by-descent graphs offer a flexible framework for imputation and both linkage and association analyses

**DOI:** 10.1186/1753-6561-8-S1-S19

**Published:** 2014-06-17

**Authors:** Elizabeth Marchani Blue, Charles YK Cheung, Christopher G Glazner, Matthew P Conomos, Steven M Lewis, Serge Sverdlov, Timothy Thornton, Ellen M Wijsman

**Affiliations:** 1 Division of Medical Genetics, Department of Medicine, University of Washington, Seattle, WA 98195, USA; 2 Department of Biostatistics, University of Washington, Seattle, WA 98195, USA; 3 Department of Statistics, University of Washington, Seattle, WA 98195, USA

## Abstract

We demonstrate the flexibility of identity-by-descent (IBD) graphs for genotype imputation and testing relationships between genotype and phenotype. We analyzed chromosome 3 and the first replicate of simulated diastolic blood pressure. IBD graphs were obtained from complete pedigrees and full multipoint marker analysis, facilitating subsequent linkage and other analyses. For rare alleles, pedigree-based imputation using these IBD graphs had a higher call rate than did population-based imputation. Combining the two approaches improved call rates for common alleles. We found it advantageous to incorporate known, rather than estimated, pedigree relationships when testing for association. Replacing missing data with imputed alleles improved association signals as well. Analyses were performed with knowledge of the underlying model.

## Background

Patterns of identity-by-descent (IBD) sharing within and across pedigrees are fundamental for the understanding of genetic variation, including its distribution, origin, and relationship to phenotype. Recent analytical and computational advances have allowed us to estimate the distribution of patterns of IBD sharing in large and complex pedigrees using the program gl_auto in the MORGAN v3.1 package (http://www.stat.washington.edu/thompson/Genepi/pangaea.shtml). These estimates are computationally intense: for example, 727 cpu minutes for family 10 (83 members) on an Intel L5427 Xeon 2.50-Gz processor. However, the resulting sampled IBD graphs can be quickly reused for several types of analysis, including genotype imputation in pedigrees [1], or obtaining [2] and refining a linkage signal [3]. At Genetic Analysis Workshop 18 (GAW18), we used these sampled IBD graphs for (a) imputation of genotypes in pedigrees compared with a "population-based" method that uses an external reference panel, (b) linkage analyses using both parametric and variance components models, and (c) association testing of both observed and imputed genotypes using two strategies to incorporate relationships between subjects.

## Methods

### Genetic map and markers

We analyzed GAW18 marker data for chromosome 3. We did not use the GAW18 sequence data because it included imputed variants, although our methods would work for sequence data as well. We obtained genetic map positions (cM) for the genome-wide association studies (GWAS) markers from the Rutgers sex-averaged interpolated positions of dbSNP Build 134 (http://compgen.rutgers.edu/maps), excluding the 116 loci missing values. Kosambi positions were converted to Haldane positions to suit assumptions made by the Lander-Green algorithm [4]. We found no Mendelian inconsistencies using Loki v2.4.7 [5] in the 65,403 markers. For linkage and association analyses, we removed markers with minor allele frequency (MAF) less than 0.05 (13,139 markers) and/or greater than 5% missing data (4,939 markers), leaving 48,892 markers for analysis.

### Phenotype and families

We began with the simulated diastolic blood pressure at time point 1 from SIMPHEN.1.csv. Given the contents of the answer key to the simulated data, we included age, sex, age*sex, and treatment in a linear regression model. The residuals are our adjusted phenotype values.

We analyzed 7 families showing evidence of cryptic relatedness in the hope of reducing genetic and allelic heterogeneity in our trait. Using all available GWAS data, we estimated kinship coefficients between all pairs of individuals in the data set using both the KING-robust [6] and REAP [7] methods to accommodate admixture, explained in detail elsewhere [8]. As is standard quality control in pedigree studies, pedigree relationships were validated by empirical estimates of kinship. Pairwise kinship coefficients exceeding those for second cousins were observed for subject pairs across families 5 to 8, 10, 21, and 25. These families were used in BEAGLE [9] imputation and SOLAR [10] association analyses described later. Family 10 was chosen for further analyses because it was the family with the strongest evidence of association to our trait [8].

### Estimation of identity-by-descent sharing

A single set of IBD graphs was used for all pedigree-based analyses. We used a subset of 351 markers with an average spacing of one marker per 0.65 cM, choosing the marker at each targeted region with the highest value of heterozygosity multiplied by the number of observed genotypes to generate IBD graphs with the program gl_auto. Markov-chain Monte Carlo sampling with a state-of-the-art hybrid sampler [11,12] allowed us to use both large pedigrees and many markers. We saved every 50th [12] of 50,000 sampled realizations of IBD graphs for chromosome 3, conditional on all observed genotypes, the genetic map, and pedigree structure [13].

### Imputation

We used the program GIGI to impute genotypes dependent on the sampled IBD graphs [1]. Imputation markers were not in the framework set used to produce IBD graphs. For each imputation marker, a set of genotypes for all subjects was sampled from the genotype probability distribution, given observed data at the imputation marker in some subjects, the sampled IBD graphs, allele frequencies, and the meiotic map. Genotype and allele probabilities were then averaged across the sampled IBD graphs. We called both alleles of a missing genotype if Pr(genotype) greater than 0.8, and otherwise called one allele if Pr(allele) greater than 0.9. Genotypes failing to meet these criteria were not called.

For comparison, we also used BEAGLE [9], which uses an outside reference panel of genotypes and population-level linkage disequilibrium to impute marker information among unrelated individuals. We compared results using three reference panels: the genotyped subjects from family 10 *and *the other families (experiment F10 + FO), only samples from family 10 (experiment F10), and the other families without family 10 (experiment FO). BEAGLE's 3,621 scaffold markers were chosen to be common (MAF >0.3) and evenly spaced (at least 0.05 cM apart). As with GIGI, we called both alleles of a missing genotype if Pr(genotype) greater than 0.8 and otherwise called one allele if Pr(allele) greater than 0.9.

**Figure 1:**
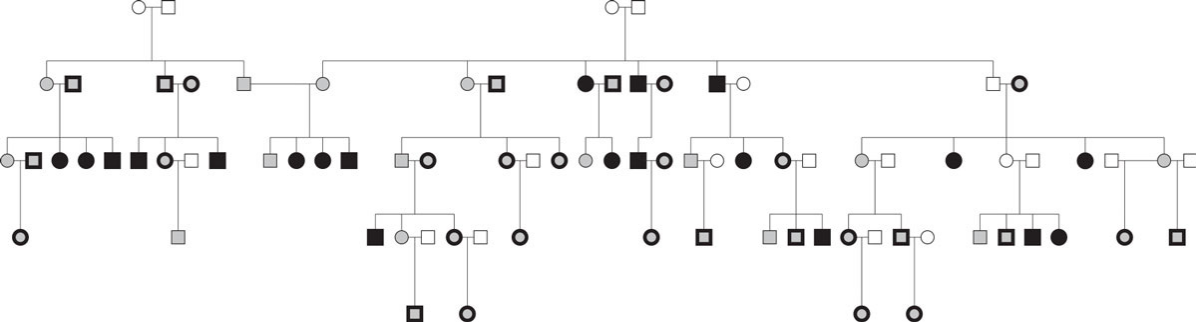
**Pedigree structure of family 10**. Filled: genotyped. Open: missing genotype data. Filled black: masked in design 1. Outlined in bold black: additional people masked in design 2.

We imputed genotypes on family 10. We masked most genotypes in a subset of subjects for evaluation of imputation metrics (Figure 1, Table 1): 20 subjects in design 1 and 49 subjects in design 2. Masked subjects were selected to preserve some "observed" genotypes in each branch of the pedigree. Imputation metrics were estimated at all imputation markers, which excluded both GIGI framework markers and BEAGLE scaffold markers. Metrics for evaluation were (a) call rate, which is the percent of alleles called, and (b) accuracy, which is the percent of alleles called correctly among called alleles. We averaged these metrics across masked variants and subjects. We define rare variants as those with MAF less than 0.05.

**Table 1:** Design of imputation experiments

		Subjects with observed marker data^1^		GIGI	BEAGLE		
Family	Subject status	Design 1	Design 2		F10 + FO	F10	FO
		Number of subjects		Number of variants observed per subject			
Family 10	Typed	44	15	65,403	65,403	65,403	0
	Masked	20	49	351^2^	3621^3^	3621^3^	36213
Others	Typed	308	308	0	65,403	0	65,403

^1^Original genome-wide association studies genotypes are observed or masked for the imputation markers as per subject status.

^2^Framework markers on chromosome 3 used to sample identity-by-descent (IBD) graphs.

^3^Dense scaffold markers used by BEAGLE to infer IBD. Scaffold markers include the framework markers.

Intrigued by the complementary data used by BEAGLE and GIGI, we evaluated a combination of their results. Using design 2 data, we first used GIGI to call both alleles if Pr(genotype) greater than 0.99 or to call one allele if Pr(allele) greater than 0.995, thus only calling alleles if essentially forced by the pedigree data. For loci with uncalled genotypes, we then used results from BEAGLE F10 + FO with call thresholds Pr(genotype) greater than 0.8 and Pr(allele) greater than 0.9. For loci with one uncalled allele by GIGI, we accepted the BEAGLE genotype if it included the single allele called by GIGI.

### Linkage analysis

We computed lod scores for family 10 and all cryptically related families at a subset of 44 positions from the IBD graphs, yielding a spacing of approximately 1 lod score per 5 cM. To obtain multipoint lod scores, we (a) used the program IBDgraph [13,14] to identify equivalence classes among the realized IBD graphs at each position [2], (b) computed likelihoods for one representative of each equivalence class at each position with the mlink program [15], and (c) computed a weighted average from the sampled IBD graphs to obtain an estimate of the multipoint lod score for the trait at each position [16].

We tested three parametric models. Model 1 is a quantitative trait locus (QTL) model with parameters defined by the single-nucleotide polymorphism (SNP) with the biggest contribution to the simulated trait variance. Because this SNP explains only 0.0229% of the simulated trait variance, model 1 tests whether we can detect a locus with a small effect size if it is modeled perfectly. Model 2 is a QTL that is the weighted average for all functional SNPs within the gene bearing the "biggest" SNP. The result is a common allele with small effect sizes and is an attempt to model the cumulative effects of several functional variants within a single gene. Model 3 is a perfectly penetrant additive locus, where affectation status indicates the subject carries the risk allele at the biggest SNP, and tests whether we could detect the SNP locus if it perfectly explained the trait variance. We compare results from the same IBD graphs with a typical variance components (VCs) lod score, as implemented by SOLAR [10].

### Association testing

We used VC analyses to investigate association with the trait of candidate covariate SNPs while accounting for correlations among related subjects. We analyzed family 10 alone, as well as all 7 families jointly. Each of the top 5 SNPs, ranked by *p*-value, identified from a half genome scan [8], was tested for association with a linear mixed model using dose of the minor allele as the fixed effect and the kinship matrix and a polygenic model as a random effect. Whereas SOLAR [10] uses the pedigree-based kinship matrix to account for relatedness, EMMAX [8] estimates the kinship matrix from the genome-wide genotype correlations. These two programs fit the same model, differing only in the source of the kinship matrix.

We also performed VC analyses with SOLAR with various combinations of imputed and observed genotype data within family 10 to evaluate the usefulness of imputed genotype data. For these analyses, we used the weighted average of genotype probabilities obtained from GIGI to provide an expected dose of the minor allele, given the observed data.

## Results

### Imputation

**Figure 2:**
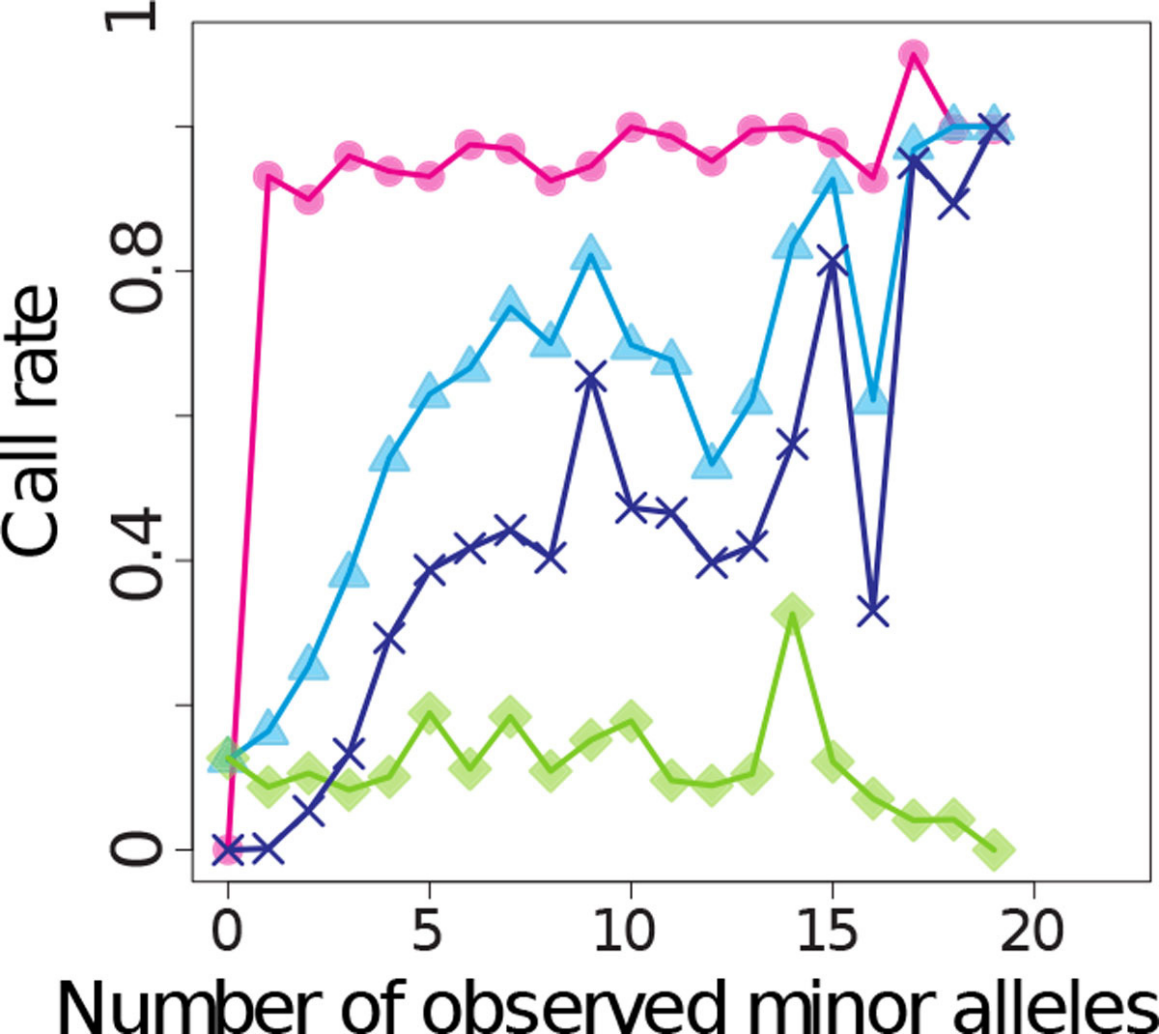
**Call rates of minor alleles for design 1 data with different imputation approaches for single-nucleotide polymorphisms with rare minor allele frequency**. Pink circles: GIGI; blue triangles: BEAGLE F10 + FO; purple crosses: BEAGLE F10; green diamonds: BEAGLE FO.

Both GIGI and BEAGLE (F10 + FO) achieved similarly high overall call rate (96.1% and 91.8%, respectively) and accuracy (99.8% and 99.1%, respectively) on the masked subjects in design 1. As demonstrated elsewhere [1,17], accuracy was high regardless of MAF. Accuracy for rare variants (MAF <0.05) was greater than 99.9% for GIGI and 99.4% for BEAGLE, and accuracy at the most common variants (MAF ≥0.45) was greater than 99.9% for GIGI and 98.9% for BEAGLE. GIGI also could impute alleles for subjects with completely missing genotype data at a call rate of 82.9%. As shown in Figure 2 for design 1 data, (a) GIGI calls more rare alleles than does BEAGLE, (b) performance differences between GIGI and BEAGLE shrank with increasing numbers of observed rare alleles within the pedigree (c) use of *only *an outside reference sample for BEAGLE was ineffective for imputation of rare alleles that may be family specific, and (d) BEAGLE was able to call a small fraction (<20%) of rare alleles that were not observed in the genotyped subjects in family 10, but only when BEAGLE had access to reference samples from other families.

**Figure 3:**
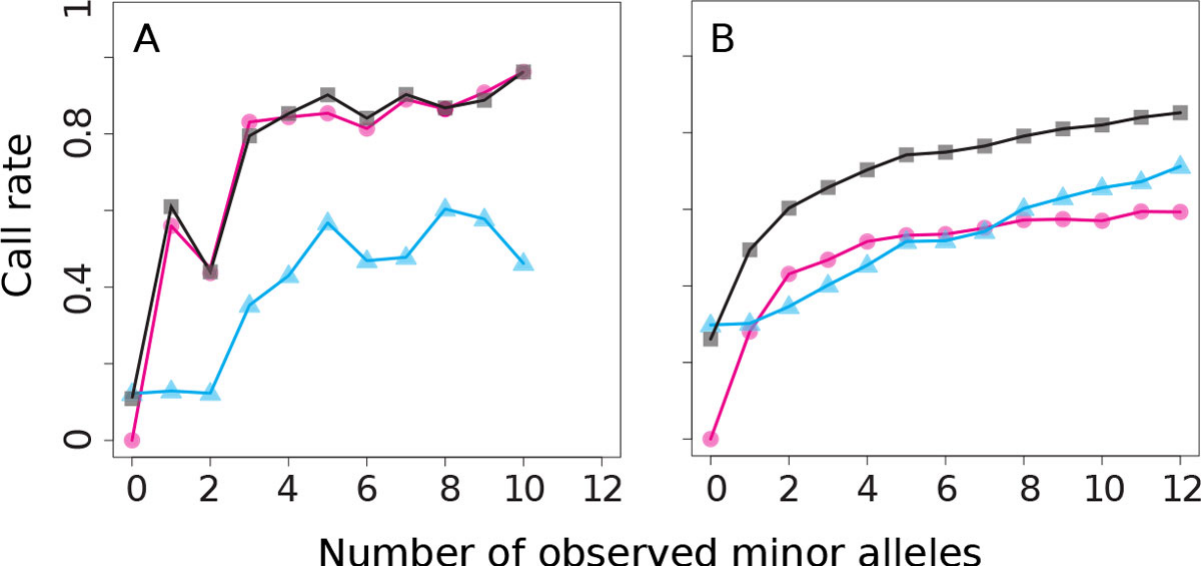
**Call rate of minor alleles for design 2 data, for single-nucleotide polymorphisms with rare (A) and common (B) minor allele frequency**. Pink circles: GIGI; blue triangles: BEAGLE F10 + FO; black squares: GIGI + BEAGLE. X-axis fixed to the maximum for rare, for consistency.

Not surprisingly, both GIGI and BEAGLE called fewer genotypes starting with the design 2 data than the more complete design 1 (call rates of 77.1% and 85.3%, respectively), with high accuracy (98.1% and 97.8%, respectively). Accuracy for rare variants was 99.5% for GIGI and 99.0% for BEAGLE, and accuracy at the most common variants was 99.9% for GIGI and 97.1% for BEAGLE. Combining GIGI and BEAGLE boosted both call rate (89.5%) and accuracy (98.6%) over the use of either alone, but only for more common variants. GIGI + BEAGLE continued to have high accuracy across MAF, with an accuracy of 99.3% for rare alleles and 98.6% for the most common alleles. Figure 3A shows little gain in call rate for the combined approach over the use of GIGI alone when imputing variants with rare alleles. In contrast, Figure 3B shows a markedly improved call rate resulting from the combined approach for SNPs with higher MAF.

### Linkage analysis

Although enough copies of the risk allele segregate within the family to generate a linkage signal if the risk allele was indeed causal (model 3, lod_max _= 5.36), this locus does not explain enough phenotypic variation within this family to provide measurable evidence of linkage (lod_max _<0.5 for models 1 and 2). VC lod score analyses [10] provided comparable results: no evidence of linkage in family 10 and an all-families' lod score near 0.2.

### Family-based association testing

Significant associations were readily detectable in both the total sample and in individual families regardless of the absence of strong positive linkage evidence (Table 2). Broadly speaking, the association tests carried out with SOLAR and EMMAX were similar to each other, with SOLAR providing somewhat stronger evidence of association. The differences between the two programs are larger for the most significant results, with the most extreme difference obtained at the causal SNP rs11711953 in family 10.

**Table 2:** Association test *p*-values from use of all available genotype data

SNP	Family 10		Seven families	
	SOLAR	EMMAX	SOLAR	EMMAX
rs11711953	7.31E-05	1.25E-03	1.57E-07	3.91E-07
rs11706549	7.31E-05	1.25E-03	1.57E-07	3.91E-07
rs6763824	2.47E-03	7.71E-03	8.93E-03	8.84E-03
rs11716779	3.78E-03	1.62E-02	2.56E-04	2.87E-04
rs17785248	3.15E-03	1.29E-02	5.51E-04	5.52E-04

*SNP*, single-nucleotide polymorphism.

As expected, Table 3 shows that the strength of association dropped when we only used genotypes for 15 subjects providing dense marker data for imputation and strengthened when we imputed genotypes into other subjects with trait data. In this data set, there was just a slight gain in the *p*-value obtained by imputing genotypes into the 20 unsampled subjects, explained by one subject who had a trait value but no observed genotype data for the 10 tested SNPs.

**Table 3:** Association test p-values from use of imputed genotype data within family 10

	N_observed _: N_imputed _: N_total _Subjects			
**SNP**	**15:0:15**	**15:48:63**	**15:68:83**	**63:20:83**

rs11711953	2.47E-02	5.24E-03	4.87E-03	6.42E-05
rs11706549	2.47E-02	5.22E-03	4.84E-03	6.42E-05
rs6763824	7.66E-02	6.33E-02	6.11E-02	3.25E-03
rs11716779	7.66E-02	6.46E-02	6.22E-02	3.14E-03
rs17785248	7.37E-02	6.15E-02	5.92E-02	2.93E-03

*SNP*, single-nucleotide polymorphism.

## Conclusions

IBD graphs provided ample opportunity to investigate relationships between individuals and between genotypes and phenotypes. Pedigree-based imputation that exploited these graphs outperformed population-based imputation for rare variants, even when the latter included family members of the subjects being imputed. We also showed that the two approaches may be combined to improve call rate and accuracy for some uses. Both parametric and VC linkage analysis failed to detect a linkage signal. Further examination revealed no cosegregation of phenotypes and genotypes at the functional variants on chromosome 3 in these families in SIMPHEN.1.csv (John Blangero, personal communication), although this was not true of the other simulated replicates. In contrast, family-based association testing with a mixed model was still able to detect association with the functional variants. We found that using the known pedigree structure in SOLAR provided similar but slightly stronger evidence for association than EMMAX, which treats subjects as unrelated but accounts for relatedness through an empirical covariance matrix. Finally, use of observed genotype data provides a stronger association signal than imputed data, although the difference between the two sets of *p*-values can be negligible. This suggests that when direct genotyping is not possible, pedigree-based imputation provides a practical and useful alternative.

## Competing interests

The authors declare that they have no competing interests.

## Authors' contributions

All authors participated in study design and analysis. EMB and EMW drafted the manuscript, and all authors edited and approved the final manuscript.
